# Lived experiences of young adults facing a recent diagnosis of cancer: A phenomenological study

**DOI:** 10.1111/hex.13793

**Published:** 2023-06-18

**Authors:** Mehraban Shahmari, Alireza Nikbakht Nasrabadi, Elaheh Rezaie, Seemin Dashti, Elhameh Nasiri, Leily Zare

**Affiliations:** ^1^ Department of Medical‐Surgical, School of Nursing and Midwifery Ardabil University of Medical Sciences Ardabil Iran; ^2^ USERN CARE (TUMS) Office, School of Nursing and Midwifery Tehran University of Medical Sciences Tehran Iran; ^3^ School of Nursing and Midwifery Tehran University of Medical Science Tehran Iran; ^4^ Hematology Department, Imam Khomeini Hospital Ardabil University of Medical Sciences Ardabil Iran; ^5^ Health Education and Promotion Department, Faculty of Health Tabriz University of Medical Sciences Tabriz Iran; ^6^ Department of Nursing Islamic Azad University Ardabil Iran; ^7^ School of Nursing and Midwifery Ardabil University of Medical Sciences Ardabil Iran; ^8^ Department of Medical‐Surgical, School of Nursing and Midwifery Tehran University of Medical Sciences Tehran Iran

**Keywords:** cancer, phenomenology, qualitative research, recent diagnosis, young adulthood

## Abstract

**Introduction:**

Young adulthood is a period of prosperity and freshness characterized by developmental achievement, which can be inhibited by various diseases such as cancer. Typically considered a terminal disease, if diagnosed in young adulthood, cancer may trigger a tremendous psychosomatic shock. The nature of facing a recent cancer diagnosis affects the whole coping process. Addressing young adults' experiences at the confirmation point of cancer diagnosis will facilitate supporting them through the early recognition of probable problems in the future. Therefore, the present study aimed to analyse the lived experiences of young adults facing a recent cancer diagnosis.

**Methods:**

This qualitative study adopted an interpretive phenomenology design. In this study, 12 patients (with an age range of 20–40) were selected using the purposive sampling method. Data collection was done through in‐depth, semistructured interviews. The data were analysed following the method proposed by Diekelmann et al.

**Findings:**

Three main themes and nine subthemes were extracted from the data: (1) spiritual detachment and then acceptance through spirituality in the form of denial and then forced acceptance, sense of guilt and spiritual help‐seeking, and anger towards God and then humbleness, (2) the shock of facing an extraordinary life shaped by disturbed role‐play and unusual lifestyle, (3) anticipatory anxiety concerning the sense of rejection, negative perspective towards future, inability to afford the costs and worries about the future of the family members.

**Conclusion:**

This was the first study providing significant insights into the experiences of young adults facing a recent cancer diagnosis. The diagnosis of cancer can shadow all aspects of young adults' lives. The findings of the present study empower healthcare professionals to provide newly diagnosed young adults with appropriate health services.

**Patient Contributions:**

To identify and recruit the participants, we explained the objectives of the present study to the unit managers either by phone or in person. The participants were approached and interviewed by three authors. Participation was voluntary and the participants received no financial contribution for their time.

## 1 INTRODUCTION

Adulthood, the period that spans the end of adolescence to the time of death, is often divided into three stages: young adulthood (the age range of 20–40); middle age; and late adulthood. Young adulthood is supposed to be the core of strength and manifestation of beauty; it is full of joy, happiness, love and hope. Young adulthood is a time of brilliance, happiness, strength, hope, effort and excitement.^1^ Although young adulthood is known to be a healthy time of life, various health‐threatening diseases such as cancer may jeopardize achieving the developmental characteristics of this stage.^1^, ^2^

Despite recent medical diagnosis and treatment advancements, cancer is still associated with pain, limitation, disability and death.^3^ Cancer is the second‐leading cause of death worldwide^4^ and the third in Iran.^5^, ^6^ According to the World Health Organization (WHO), cancer caused 10 million deaths (one per six deaths) worldwide, with an average age of 72 in 2020.^4^ Cancer is still considered a disease that people avoid even talking of, associating with concepts such as evil enemy, unpredictable, indestructible, death and suffering.^3^ Cancer, commonly known as an incurable and terrible disease, is equivalent to death. Therefore, facing cancer diagnosis shocks young adults^7^, ^8^, ^9^ and affects their physical, mental and emotional health and developmental tasks.^10^

Whatever the nature of this exposure would be, it affects the course of the disease afterward. Healthcare providers as witnesses of this phenomenon should be knowledgeable in providing the required care services. Exploring young adults' experiences at the stage of diagnosis confirmation will facilitate the provision of help through early recognition of potential problems. Studies in this area, especially in Iran, are limited. Reaction to cancer diagnosis seems context‐based. Therefore, the present study aimed to explain the lived experiences of young adults facing recent cancer diagnosis, over the last 1–3 months. Using phenomenology‐driven design in this study enables the researchers to understand the subjective meaning of facing a cancer diagnosis in young people. This perspective also provides a proper understanding of what impact this experience may have on young people's lives. Furthermore, qualitative data analyses will explain their perceptions of experiencing a recent cancer diagnosis.

## 2 METHODS

This qualitative study was conducted using an interpretive phenomenology from November 2021 to April 2022 and reported based on a set of consolidated criteria for reporting qualitative research (COREQ) checklist.^11^ The research environment was the outpatient chemotherapy department and haematology ward of the largest hospital in one of the northwestern cities in Iran.

### 2.1 Participants

The purposive sampling method was used to select the participants. The inclusion criteria were (a) adults aged between 20 and 40, (b) a definite diagnosis of cancer over the last 1–3 months, (c) willingness to communicate and express real‐life experiences and (d) absence of any known psychotic illness based on medical documents. The participants who were selected had diverse characteristics in gender, education, occupation and definitive diagnosis of different cancer types to achieve the maximum level of diversity and richness of information. The first and second authors contacted the potential participants. These two researchers scheduled face‐to‐face interviews. Fourteen patients were contacted gradually. Two were unwilling to share their experiences. Finally, 12 participants agreed to be interviewed.

### 2.2 Data collection

Three authors conducted semistructured, in‐depth interviews under the supervision of the senior author. Two of the interviewers were PhD students in Nursing and intensive care/emergency nurses for 14 years, trained in doing qualitative research and conducting interviews. The other interviewer was a clinical nurse with 7 years of experience in the haematology ward. The nurse was trained by the other two interviewers on how to conduct interviews in qualitative research in several sessions before the study. Therefore, the interviewers were familiar with the unit managers and asked them to introduce the eligible patients. The supervisor of this study was a faculty member in Nursing with prior experience in conducting qualitative research. The interview questions were made based on the literature review^12^, ^13^, ^14^ and the expert opinions of the researchers. At the beginning of the interviews, demographic questions were asked, and then continued with more specific questions related to the purpose of the research (Supporting Infomation: Box 1). All interviews were audio recorded with the consent of the participants. A total of 15 interviews were done with 12 participants to reach data saturation,^15^ of which three were supplementary to acquire rich and in‐depth data. The senior investigator assessed saturation which is presented in authors section. In general, all interviews were conducted in person in a quiet place without the presence of others. The mean, minimum and maximum duration of the interviews were 56, 45 and 80 min, respectively.

### 2.3 Data analysis

The goal of analysing phenomenological data is to ‘transform lived experience into textual expression and thus gain essence’.^16^ Based on Heideggerian beliefs Diekelmann et al.^17^ devised a step‐by‐step process of analysing narrative text. The analysis is typically done by an interpretive team and involves seven steps: (a) reading all interviews to gain an overall understanding; (b) writing interpretive summaries and coding them; (c) analysing emerging themes with the team; (d) returning to the text to confirm the analysis; (e) comparing and contrasting texts to identify common meanings; (f) identifying patterns that link the themes; and (g) eliciting basic pattern and final draft by the interpretive team.

In this study, data analysis started by repeatedly listening to the recordings to extract the overall idea and then continued according to the abovementioned seven steps. Data management was done using MAXQDA10 software.

### 2.4 Trustworthiness

The following strategies were carried out to achieve rigour and trustworthiness.^16^ Credibility was obtained through prolonged engagement with the participants and data, and the allocation of enough time to data collection. Dependability was ensured by examining the consistency between the quotes and the codes/subthemes that emerged from the research team and two external observers familiar with qualitative research. Confirmability was ensured by presenting quotes as precisely as extracted from the interviews. Transferability was enhanced by detailing the entire research process, the characteristics of the participants and the research context.

### 2.5 Ethical considerations

The ethics committee approved this study, and permission was obtained from the relevant authorities. The purpose of the study and relevant information were clarified. The researchers also highlighted the voluntary nature of participation, its confidentiality and the right to withdraw. Verbal and written consent was obtained before the interview. Permission to record the interview with an audio recorder was also obtained.

## 3 FINDINGS

The participants aged between 20 and 40 were predominantly female (Table 1). Three main themes and nine subthemes were extracted from the data reflecting the lived experiences of the young adults facing the recent diagnosis of cancer (Table 2).

**Table 1:** Demographic characteristics of the participants.

Participants	Sex	Age	Marital status	Education	Occupation	Children	Cancer type
1	Female	26	Single	Bachelors	Housewife	No	Breast malignancy
2	Female	35	Married	Bachelors	Housewife	Yes	Gum malignancy
3	Male	30	Single	Bachelors	Employee	No	Colorectal malignancy
4	Male	40	Married	None	Freelancer	Yes	Colorectal malignancy
5	Female	36	Married	Bachelors	Employee	Yes	Bone marrow
6	Male	38	Married	Associate degree	Manual worker	Yes	Colon
7	Female	37	Married	None	Housewife	Yes	Lymphoma
8	Female	39	Married	Diploma	Housewife	Yes	Breast malignancy
9	Female	40	Married	None	Housewife	Yes	Leukaemia
10	Female	40	Married	None	Housewife	No	Uterus
11	Female	39	Divorced	None	Housewife	No	Uterus
12	Female	38	Married	Primary school	Housewife	No	Breast malignancy

**Table 2:** Overview of the main themes and subthemes extracted from the data.

Main themes	Subthemes
Spiritual detachment and then acceptance through spirituality	Denial and then forced acceptance
	Sense of guilt and spiritual help‐seeking
	Anger towards God and then humbleness
The shock of facing an extraordinary life	Disturbed role‐play
	Unusual lifestyle
Anticipatory anxiety	Sense of rejection
	Negative perspective towards future
	Inability to afford the costs
	Worries about the future of family members

### 3.1 Spiritual detachment and then acceptance through spirituality

Facing a recent cancer diagnosis is the most traumatic event in young people that leads to experiencing contradictory feelings of spiritual detachment and then acceptance through spirituality in the form of denial and then forced acceptance, sense of guilt and spiritual help‐seeking, and anger towards God then humbleness.

#### 3.1.1 Denial and then forced acceptance

According to more than two‐thirds of the participants facing a sudden cancer diagnosis, they experienced reciprocal feelings from forced acceptance to the denial of the disease. It was unbelievable to accept having such an incurable disease in young adulthood. Therefore, they insisted that there must have been a mistake or this could not have been true. They even hide the situation from friends and relatives.

When I first discovered that I had cancer, I cried so much and became so upset that I fell asleep. I kept saying that something must have happened; it could not be correct; we had no history of such a disease in our family; why me? (Participant 10)

Meanwhile, the participants stated that they had no choice but to accept the disease inevitably. Dealing with the disease would calm them, make it easier to bear the pain and discomfort, and increase their hope. Also, with knowledge of the recovery process of similar patients, the participants feel optimism.

Over time, after being visited by many physicians in different cities, and being tested several times, the disease was confirmed. I had no choice but to give up and accept it. (Participant 1)

#### 3.1.2 Sense of guilt and spiritual help‐seeking

Guilt was another paradoxical emotion that some participants experienced. According to them, being diagnosed with cancer is a punishment from God for committing sins, so they blamed themselves.

In the beginning, I was always crying, thinking about the bad things I did and why I got sick. This must be because of my sins. (Participant 5)

On the other hand, more than half of the participants stated that they tried to accept the disease and endure the exhausting process by asking God through praying, reciting the Quran and giving vows. They referred to their close relationship with God as a source of their peace. In case of experiencing the alleviation of symptoms, they were grateful to God.

At this time, you feel closer to God and put more trust in him. You talk to God faithfully as your trust in God has increased tremendously. (Participant 2)

#### 3.1.3 Anger towards God and then humbleness

Anger towards God by complaining, fighting and being unkind to God is another paradoxical emotion of young adults since they believed that God, as the absolute source of power, could have prevented this terrible and overwhelming disease.

As soon as I found out, I cried, screamed, and kept saying, ‘God, why me? I always help others; I was always looking for good deeds; why did you let me get sick? Aren't you God? Don't you have the power? Why do you like to hurt me?’ (Participant 12)

Some 50% of participants stated that they experienced changes in their tendencies from materialism to spirituality. Since they consider themselves closer to death, they desire simple living, modesty and gentleness when interacting with others.

I was very arrogant, but now I am not anymore. Ever since getting the disease and feeling closer to death, I have learned to have a simpler lifestyle and treat others gently. (Participant 2)

### 3.2 The shock of facing an extraordinary life

More than half of the participants interpreted the recent diagnosis of cancer as the shock of facing an extraordinary life, which triggered their disturbed role‐play and unusual lifestyle.

#### 3.2.1 Disturbed role‐play

As the age of the participants (20–40 years) implies, independence is an inseparable concept in all dimensions of their lives. After facing the disease and the diagnosis of cancer, the patients felt that this disease, which is both traumatic and dreadful in their minds, threatens their independence. The loss of independence was so difficult and exhausting that most participants stated they preferred death over dependency on others. Being a burden was one of their main concerns. That is why they made all their efforts to maintain their independence.

I am worried about being dependent on others and burdening them. I pray to God and ask not to make me dependent on anyone. I prefer death to be a burden on others. (Participant 4)

Almost all patients mentioned the significance of their independence in playing different roles in their personal and social lives. A lack of identity in playing a role is one of the crucial experiences after being diagnosed with cancer. Almost all patients further highlighted experiencing impairments in playing different roles such as the role of a mother and a wife. Even considering the recent diagnosis of the disease (over the last 1–3 months), this disease prevented them from going to work and caused concerns about staying home.

At the moment, I am going to the doctor. I do not go to work. The other day, my wife told me my son works with my car. Well, our life has turned into a mess, and I do not know what is going to happen. (Participant 4)

#### 3.2.2 Unusual lifestyle

A sudden encounter with a cancer diagnosis in young adulthood means disturbances, nightmares, a sense of anxiety and extreme apprehension about the disease which leads to departing from the ordinary lifestyle.

Accordingly, cancer is considered a dreadful disease that leads to a feeling of extreme change in the lives of young people. To almost all participants, fear and worry about the disease and the consequences of its treatment have disturbed their whole lives. It caused harsh conditions for the patients and their families. This means that they cannot live their usual lives. They thought of cancer as a life with severe complications. They stated that having learned about the diagnosis and treatment of their disease, they experienced some physical reactions such as heart palpitations, diarrhoea, vomiting, weight loss, hair loss, weakness and lethargy, low blood pressure, bone pain, weakened immune system and even a case of foetal death. These factors led to the deterioration of the patients' living conditions. These factors caused some participants to search for any method to relieve symptoms and use alternative treatments such as herbal teas and even go to fortune tellers, exorcists, and so on.

Since the diagnosis of the disease, our whole life has changed, and even the whole system of my body has been messed up. I had heart palpitations. I got digestive symptoms. I feel that from now on, my life will be full of complications and different from others. (Participant 8)

### 3.3 Anticipatory anxiety

Another theme evident in nearly all participants' statements was excessive worries about potential future events such as a sense of rejection, negative perspective towards the future, inability to afford the costs and worries about the future of the family members as the sub‐themes.

#### 3.3.1 Sense of rejection

More than half of the participants stated that they believe cancer is not a disease that can be cured quickly. They even considered it a terminal disease that may cause them to be worried about being rejected by their partners, families and friends.

They also expressed that facing a cancer diagnosis and upon inquiry about the complications of the disease and its treatment, they felt apprehensive about the judgement of others about the changes in their appearance. Moreover, the patients stated that after starting the treatment, the mental picture of their body was impaired following hair loss and severe weight loss. Subsequently, they felt embarrassed and avoided attending gatherings, and even during the coronavirus disease 2019 pandemic, they expressed satisfaction with the ban on holding gatherings. Some patients who underwent surgery expressed concern about the scar on the surgical site, and those who underwent mastectomy complained about limitations on wearing their favourite clothes.

I think about how my hair will fall out when I do chemotherapy and how I will look in public, and I feel like I'm going to be embarrassed; I think about these things all the time. (Participant 6)

The altered body image reinforced the sense of rejection which was specifically more common among married women who were worried about their husbands leaving them at any moment.

You know, sometimes I think that if it takes too long and I fall out of shape, my husband will go to someone else. Of course, he is not that kind of man, but I always think about this. I pray to God that this will not happen to me. (Participant 7)

#### 3.3.2 Negative perspective towards the future

One of the patients' concerns after facing a cancer diagnosis is the feeling of disappointment due to the uncertain future of the disease. They stated that they had doubts about recovery because they feared the incurable nature of the disease and were worried about its treatment that would be prolonged mainly due to the lack of medicine. Furthermore, the participants expressed feelings of inability to adapt to the disease. In some cases, due to the public's opinion about the incurability of the disease, they felt the imminent death. They even thought of suicide due to being in an unknown situation, losing their spirit and feeling negative towards recovery.

Well, the name of this disease is scary, and people do not even dare to talk about it. All I think about is that the end of this disease is death, so I am not sure of being completely cured. (Participant 8)

#### 3.3.3 Inability to afford the costs

Another main concern of all patients is treatment costs since it has been relatively accepted in society that the treatment of this disease is costly. Concerns about affording treatment were mostly reported. Besides, work‐related difficulties arise from extended leaves, which may lead to an insurance cut‐off. These factors caused the patients not to follow their treatment seriously.

I live in a village and sell several cows every time I come here for treatment. While I am away, my sister is responsible for the housework and the cows. I always asked God whether I can pay for my treatment. (Participant 11)

#### 3.3.4 Worries about the future of the family

Some 50% of participants, especially the married ones, stated that upon encountering the sudden diagnosis of cancer, they experienced worrying about the future of their family and children, not seeing their children's weddings, missing their children and being away from them. On the other hand, most of the participants stated that this kind of distress aggravated their confusion. These were the main factors in pursuing treatment.

All I'm saying is, God, for the sake of my children's future, help me get well. At least give me a chance to raise my children because I do not know what will happen to them if I die. (Participant 8)

With this disease, my life has completely changed. All family members were shocked and confused. My family tries not to say anything, but I feel that they are worried. My disease has destroyed the whole family. (Participant 9)

## 4 DISCUSSION

This study provides significant insights into the aim of the study by explaining the lived experiences of young adults facing a recent cancer diagnosis. Since the study has been done 1–3 months after being diagnosed with cancer, this short time period can provide great insight into the immediate outcomes of being diagnosed with cancer. Three main themes were extracted: spiritual detachment then acceptance through spirituality, the shock of facing an extraordinary life and anticipatory anxiety.

Spiritual detachment and then acceptance through spirituality was the first main theme, which showed the contradictory feelings and behaviours of the participants. When young patients faced the sudden diagnosis of cancer, they experienced different mental and emotional reactions, including denial and then forced acceptance, a sense of guilt and spiritual help‐seeking, and anger towards God and then humbleness on both sides of the spectrum. Various studies, in line with the findings, reported that upon facing a cancer diagnosis, people show different reactions such as denial, astonishment, sadness, blame, anxiety, fear, worry, despair, anger, guilt and loneliness along with some positive emotions.^18^, ^19^ In the current study, young people suffering from various types of cancer considered spiritual help‐seeking as one of the pleasant experiences essential for adapting to the conditions caused by the disease. In a similar study, Mehrabi et al.^20^ referred to the reliance on religion and spirituality as the primary source of psychological support. In another study on women with breast cancer, also religious beliefs and practices were stated as the keys that helped patients cope with the disease.^21^ While another study mentioned engaging in mindfulness activities like art therapy programs and yoga as a coping strategy in young women with metastatic breast cancer.^22^ On the other hand, Curtis et al.^18^ reported both internal resources and external ones necessary to cope with cancer. In the present study, patients handle the situation just by taking advantage of religious beliefs. This may be for the differences in the participants' age, the average age of participants of that study was 53. The religious background of society can influence coping skills as well. Also, the time span of treatment and diagnosis are the other two influencing factors.

Participants were shocked by facing an extraordinary life manifested through disturbed role‐play and experiencing an unusual lifestyle. In contrast, another study investigating young women with metastatic breast cancer reported that participants experienced higher shock and impaired quality of life compared to their first diagnosis.^22^ Most participants experienced vital concerns about the loss of independence and subsequent disruption in role performance, which were not compatible with the development of independence in young adults. At a young age, there are specific developmental tasks such as physical, psychosocial, cognitive, moral and spiritual ones. At this stage of life, the musculoskeletal system is well‐developed. Psychological and social development includes being independent of parents, having a realistic self‐image and self‐love, managing one's life, interacting with the family, dealing with the tensions of change and growth, establishing excellent and intimate relationships with others, getting married, taking emotional, social and economic responsibilities, and living a healthy life.^1^ In line with the current study, in a qualitative phenomenological study on young adults with thyroid cancer, Smith et al.^19^ reported, that young people experienced a loss of youthful immunity that contrasted with a sense of growth and change in life. Additionally, in other study studies, the diagnosis of breast cancer in women and the subsequent surgery led to experiencing identity disorders in the affected women so that they lost their womanhood.^18^, ^21^ For many patients, cancer diagnosis and treatment could be considered as a highly stressful experience that makes individuals vulnerable to negative long‐term psychological consequences including emotional distress, depression, anxiety, sleep problem, fatigue and reduced quality of life.^23^, ^24^

In this study, the young people also experienced an unusual life along with the confusion that dominated the lives of the patients and their families. Most participants stated that imagining life with complications led to experiencing nightmares, anxiety and extreme apprehension due to the shock of illness and loss of life. In line with the present study, a range of emotions, including experiences of shock and vulnerability, have been reported in young people with thyroid cancer.^19^

For most of the participants, cancer was equivalent to excessive worry about bad happenings in the future, indicating concern about the change in the mental image of the body resulting in fear of rejection, negative perspective towards future, fear of not being able to provide financial expenses and concern about the future of the family. The present study showed that young people were so worried about physical changes in their bodies, such as hair loss, weight loss, colour change and so on, that they avoided attending parties, meeting others and choosing their clothes. Other studies have similarly demonstrated that changes in body image, feeling ashamed of being in public and dealing with negative attitudes and stigma are the main challenges that women with breast cancer may experience.^20^, ^21^, ^25^

One of the bitter experiences of some participants, especially the married ones, was their concern about the future of their family and children, which was stated as one of the main factors in pursuing treatment. In the same way, in other studies, concerns about the future of children and family members, as well as the acceptance of the spouse, is reported to be the most important psychological and emotional reaction of women with cancer. This concern is also one of the main factors in starting the treatment_._ ^21^, ^24^

The patients also had negative perspectives towards the future when thinking about the uncertain future of the disease and the fear of being incurable, being in an unknown situation and being hopeless about recovery that may activate the thoughts about suicide in some participants. In other studies, fear of death and uncertainty about the future has been reported as the main concerns for people with cancer.^13^, ^21^ On the other hand, in many studies, having an optimistic attitude, including the hope of recovery and returning to a healthy life, has been reported as a very motivating resource for adapting to cancer at different ages_._ ^21^, ^26^, ^27^

Cancer treatment has caused an enormous cost to families and societies.^20^ As in the current study, most young people with cancer expressed financial problems as a primary concern for continuing treatment. Hamid et al.^21^ similarly highlighted financial problems as one of the challenging issues for women with cancer in India. Hence, the patients may delay the onset of treatment due to financial problems. The present study differs from the abovementioned study in terms of involving both genders of young adulthood. However, in both countries, due to the high cost of cancer treatment and the health insurance problem, the concern about treatment costs is reported to be one of the main factors preventing patients from pursuing treatment. These findings are in contrast to the results of a study by Williams and Jeanetta,^25^ who reported that women with cancer in the United States do not face financial problems due to their health insurance support. McNeil et al.^28^ examined the financial impact of cancer, the use of income support and parental caregiving (6–24 months after diagnosis) in adolescents and young adults aged 15–25 in Australia. In line with our study, they reported that more than half of the participants had financial problems due to cancer. Although adolescents, parents and young adults reported that financial support was essential to them during and after cancer treatment, they encountered financial issues triggered by direct medical and indirect expenses.

## 5 LIMITATIONS

Our study is affected by some limitations. For instance, since this study is qualitative, the findings may not be directly generalized to other communities or contexts. Considering the fact that cancer is a critical disease that individuals feel unpleasant to talk about, lengthy and exhausting interviews were not unexpected. Moreover, male patients were less willing to express their experiences; thus, fewer males participated in the study.

## 6 CONCLUSION

By extracting the mentality and experiences of young people facing a recent cancer diagnosis, this study has added new findings to the existing body of knowledge regarding the difference in culture and beliefs of these patients. The findings showed that cancer diagnosis in young people significantly affects them because as the patients admitted, it had been the most traumatic experience in their lives. Emotional turmoil, including anger at God, denial of the disease and extreme embarrassment were among the common immediate reactions of the young patients. However, they accepted the reality and used different methods to deal with their illness over time.

### 6.1 Implications and suggestions

Relying on the findings of this study, healthcare providers can facilitate offering health services to young adults who have recently been diagnosed with cancer by concentrating on the early recognition of possible future problems. Moreover, exploring the individuals' perceptions of their disease in the early stages may reduce the impact of the diagnosis. According to the literature, it can promote coping and compatibility with diagnosis through interventions such as providing appropriate information, gaining membership in peer groups, having access to the stories of other cancer patients, improving communications with healthcare professionals^18^, ^19^ and reliance on religion and spirituality^20^, ^21^ as well as mindfulness and meditative activities.^22^, ^29^ Furthermore, the policymakers working in the healthcare system can benefit from the findings of the present study to formulate some health insurance plans for the purpose of covering the costs of young people with cancer.

Considering the fact that this research study was conducted on the acute phase of the disease, it is suggested to investigate and compare the views of these patients with those who have survived cancer from different personal and social perspectives. Testing these findings in the framework of quantitative research helps to increase the reliability and generalization of the results and their applicability. Given that, the experience of an unusual lifestyle was a vital concern for participants. However, there is less research on the quality of life of young adults facing a recent cancer diagnosis. Thus, it is worth conducting research on the quality of life of young adults facing a recent cancer diagnosis for planning to promote their quality of life.

## AUTHOR CONTRIBUTIONS

Mehraban Shahmari and Leily Zare contributed to the conception and design, collection of data, analysis, and interpretation of data, drafting of the article and reviewing and editing the original draft. Alireza Nikbakht Nasrabadi contributed to the analysis and interpretation of data, review and editing of the final draft, final approval of the version for publishing and general supervision of the research group. Elaheh Rezaie contributed to the collection of data and drafting of the article. Seemin Dashti and Elhameh Nasiri contributed to drafting the article and reviewing and editing the original draft.

## CONFLICT OF INTEREST STATEMENT

The authors declare no conflict of interest.

## ETHICS STATEMENT

This study was done in full accordance with the ethical principles of the Declaration of Helsinki. The proposal for this study was approved by the Ethics Committee of the Research Council of TUMS with the code: IR.TUMS.VCR.REC.1399.594. To participate in the study, informed written consent was obtained from the participants.

## Supporting information

Supporting information.

## Data Availability

The data supporting the findings could be made available by the corresponding author. The data are not publicly available due to privacy or ethical restrictions.
