# Supplementary material for: Lived experiences of young adults facing a recent diagnosis of cancer: A phenomenological study
Source: Health Expect. 2023 Jun 18;26(5):1874–82. doi: 10.1111/hex.13793 (PMC10485348; doi:10.1111/hex.13793)
Supplement: Supplementary file 1 — Supporting information. [file HEX-26--s001.docx]

**Box 1. Semi-structured interview guide**

1. How did you become aware of your disease?
2. How did you feel when you were diagnosed with cancer?
3. What was your experience like?
4. How would you describe it?
5. What happened to you?
6. What were you thinking about?
7. How did you manage to cope with reality?
8. What changes have happened in your life since you were diagnosed with cancer?
9. Can you explain more about this?
10. Is there anything else you would like to explain?
